# Assessing growth mindset in undergraduate medical students: translation, cross-cultural adaptation, and evaluation of the Chinese version of the implicit theories of intelligence scale

**DOI:** 10.3389/fmed.2026.1969574

**Published:** 2026-10-29

**Authors:** Ying Xie, Wei-Han Hong, Gang Chen, Chan Choong Foong

**Affiliations:** 1 Shengli Clinical Medical College of Fujian Medical University, Department of Hematology, Fujian Provincial Hospital, Fuzhou University Affiliated Provincial Hospital, Fuzhou, China; 2 Medical Education and Research Development Unit (MERDU), Faculty of Medicine, Universiti Malaya, Kuala Lumpur, Malaysia; 3 Shengli Clinical Medical College of Fujian Medical University, Department of Endocrinology, Fujian Provincial Hospital, Fuzhou University Affiliated Provincial Hospital, Fuzhou, China

**Keywords:** mindset, medical student, translation, cross-cultural adaptation, reliability, validity

## Abstract

**Objectives:**

Cross-cultural equivalence translations of the widely used Implicit Theories of Intelligence Scale (ITIS) into Chinese are limited. Past research has focused on Western educational contexts, and Eastern cultural perspectives have been overlooked. This study aimed to translate and validate the Chinese version of the ITIS, and assess the growth mindset in undergraduate medical students.

**Methods:**

This study was conducted among final-year undergraduate medical students in Fujian, China. Following forward and back-translation of the Implicit Theories of Intelligence Scale (ITIS), cognitive interviews were conducted with students to assess the clarity and comprehensibility of the translated items, followed by an online survey distributed through medical schools. Construct validity, convergent validity, and discriminant validity were evaluated using confirmatory factor analysis. Internal consistency was assessed using Cronbach's alpha and composite reliability (CR), while test–retest reliability was evaluated separately for the Fixed Mindset and Growth Mindset dimensions using the intraclass correlation coefficient (ICC).

**Results:**

A total of 444 responses were obtained. Confirmatory factor analysis supported a two-factor structure with satisfactory model fit (*χ*^2^ = 52.293, *df* = 17, CMIN/DF = 3.076, CFI = 0.984, NFI = 0.977, RFI = 0.962, IFI = 0.984, TLI = 0.974, RMSEA = 0.068, SRMR = 0.059). Standardized factor loadings were satisfactory, while CR values were 0.908 for Fixed Mindset and 0.910 for Growth Mindset, and AVE values were 0.713 and 0.717, respectively, supporting convergent validity. Discriminant validity was supported by the Fornell–Larcker criterion, AVE values exceeding the corresponding maximum shared variance values, and HTMT ratios below 0.90. Test–retest reliability differed between the two dimensions: the Fixed Mindset dimension demonstrated moderate temporal stability (ICC = 0.662, 95% CI: 0.443–0.806), whereas the Growth Mindset dimension showed limited temporal stability (ICC = 0.159, 95% CI: −0.158–0.446). The mean scores for Fixed Mindset items ranged from 2.64 (SD = 1.09) to 2.86 (SD = 1.56), whereas those for Growth Mindset items ranged from 3.81 (SD = 1.16) to 3.96 (SD = 1.21), indicating relatively stronger endorsement of fixed-mindset beliefs in this sample.

**Conclusions:**

The Chinese version of the ITIS is a valid and reliable instrument for assessing the intelligence mindset of medical students.

## 1 Introduction

Mindset refers to an individual's underlying beliefs and assumptions about the nature of human attributes, such as intelligence, talent, and personality. It reflects how individuals perceive themselves and the world, thereby influencing their attitudes, behaviors, and responses to challenges and opportunities (1). Intelligence mindset refers to an individual's belief about the nature of intelligence and its plasticity. Those with a fixed-intelligence mindset view intelligence as a rigid, unchangeable trait, whereas those with a growth-intelligence mindset view it as a dynamic, developable attribute that can be cultivated over time (1). The significance of the intelligence mindset lies in its profound impact on individuals' learning, performance, and personal development (1–3).

Research has consistently demonstrated that individuals with a growth-intelligence mindset are more resilient, motivated, and adaptable in the face of challenges. They are also more likely to embrace learning opportunities, persist through difficulties, and achieve higher levels of success (4, 5). In contrast, those with a fixed attitude shy away from challenges and give up when faced with difficulties, which leads to lower grades in school (6, 7). Developing a growth-intelligence mindset has been linked to improved academic performance as well as career advancement and overall well-being (3, 8, 9).

Early research has mostly focused on the growth mindset of primary school (10, 11) and adolescent students (12–14). Comparatively fewer studies have explored mindset development in undergraduate students (15–18), including medical students (19, 20). Notably, emerging evidence in health profession education has begun to bridge this research gap by examining mindset-related outcomes.

Previous studies suggest that a growth mindset can enhance medical students' motivation, resilience, and perseverance. It encourages them to seek and accept feedback, view failure as a learning opportunity, and adopt a goal-oriented approach that emphasizes the learning process (21–23). Medical students likely face greater challenges and stress than students from other majors, especially when they begin clinical practice. During the clinical year, medical students shift from learning through lectures in a classroom setting to learning from real patients' issues in a clinical setting. Consequently, they must engage in self-reflection and change at a faster pace to close gaps in their knowledge and skills (24). Meanwhile, they undergo challenging clinical experiences, such as first-time encounters with death and seriously ill patients or disharmony caused by conflicts between their internal ideals and stated or implied standards from their teachers or the external environment (24, 25). Medical students also face various other difficulties, such as increased workload, insufficient study time, and limited feedback and supervision from clinical teachers (26). Whether students can effectively cope with this high-pressure study depends on their learning motivation, beliefs, and behaviors (23, 27).

Because mindsets impact medical students, methods for accurately measuring them have become a central research focus. The Implicit Theories of Intelligence Scale (ITIS), developed by psychologist Carol S. Dweck in 1999, was designed to measure an individual's intelligence mindset (28). It has been widely used in research and educational settings to assess individuals' beliefs about intelligence and their impact on behavior and outcomes. Previous studies have shown that the ITIS has good reliability and validity (22, 29–31).

However, to our knowledge, studies have primarily focused on the Western context, whereas translated Chinese versions of the ITIS are limited (19, 21). The two existing translated Chinese versions of the scale have not adopted a cross-cultural equivalence translation (32, 33). East and West have different cultures and language systems, and translations may be misleading in the context of other cultures (34). Therefore, if the intrinsic meanings of words in different cultures are not considered during translation, the original meaning of the questionnaire may be distorted, thereby affecting the accuracy of the questionnaire when measuring the mindset among Chinese participants.

To ensure a universalist approach to cross-cultural research, Herdman *et al*. (35) developed a model of equivalence for the cross-cultural translation process. The model emphasizes five types of equivalence: (1) Conceptual Equivalence is achieved when questionnaires share the same underlying idea in both cultures, namely, in terms of the domains included and the emphasis placed on different domains; (2) Item Equivalence is achieved when items are equally relevant and accepted in both cultures and estimate the same parameters on the latent trait being measured; (3) Semantic Equivalency refers to transferring meaning between languages and achieving a similar effect on respondents in different languages; (4) Operational Equivalence refers to the possibility of using a similar questionnaire format, instruction mode of administration, or measurement methods. When these factors have no effect on the outcomes, equivalence is reached; and (5) Measurement Equivalence ensures that different language versions of the same instrument achieve acceptable levels of reliability, responsiveness, and construct validity. Because each of these different types of equivalence is important to the process as a whole, it is crucial to summarize the process and findings with a final type of equivalence known as Functional Equivalence. The model has been shown to enhance the validation of scales across different cultures due to its emphasis on semantic modifications to ensure relevance, acceptability, clarity, and minimal cultural adaptation (36, 37). Given the importance of intelligence mindset measurement for Chinese medical students and the necessity for a cross-cultural equivalence translation of the ITIS, we translated and validated the Chinese version based on Herdman's framework.

## 2 Methods

### 2.1 Participants and setting

In China, the clinical medical program spans five years. The initial three years are dedicated to theoretical coursework at the institution. In the fourth year, students commence clinical observations, and in the fifth year, they are fully immersed in clinical practice. Therefore, fifth-year students were chosen for our study, because they had undergone both preclinical and clinical training and, thus, possessed a comprehensive understanding of medical training.

The inclusion criteria were: (1) currently enrolled as an undergraduate clinical medicine student in Fujian Province; and (2) being in the fifth year of undergraduate medical education. Students were excluded if they were enrolled in a non-clinical medicine programme, were not in their fifth year of study.

Our study included final-year undergraduate medical students from medical colleges in Fujian Province, namely Fujian Medical University, Fujian Chinese Medical University, Xiamen University, Xiamen Medical College, and Putian Medical College, with an estimated population of 1,800 in all the institutes combined.

Based on an estimated population of approximately 1,800 eligible students, the minimum effective sample size for our study was calculated to be *n* = 317, using the online sample size calculator RaoSoft® (http://www.raosoft.com/samplesize.html), with a confidence level of 95% and a margin of error of 5%.

### 2.2 Ethics approval and consent to participate

This study was approved by the Ethics Committee of Fujian Provincial Hospital on on September 28, 2023 (Approval No.: K2023-09-004). Furthermore, the study was conducted according to the principles of the Declaration of Helsinki (version 2013). Before answering the online questionnaire items, respondents were shown an informed consent page. They were told that by clicking “the next page,” they were providing their consent to participate in this research. Hence, digital informed consent was obtained from all participants prior to them answering the questionnaire. The dates of informed consents obtained were different, depending on respective dates (and time) each respondent answered the questionnaire.

### 2.3 The implicit theories of intelligence scale

The ITIS was originally developed in English by psychologist Carol S. Dweck (38). It has eight items and encompasses two domains: fixed and growth mindsets. The initial four items appraise a fixed mindset, whereas the subsequent four probe a growth mindset. Each item offers six response options, ranging from 1 (strongly agree) to 6 (strongly disagree), with lower scores indicating the dominant mindset of participants. For each dimension, the scores of the four corresponding items were summed and divided by four to obtain the mean subscale score.

### 2.4 Translation process

The translation of the ITIS adopted Herdman's model of cross-cultural translation to achieve conceptual, item, semantic, operational, and measurement equivalence (Figure 1). Before the translation of ITIS, the first author reviewed the description of “intelligence” in Chinese literature to determine whether it had connotations similar to the concepts in the questionnaire. The ITIS was translated into Chinese according to Geisinger's guidelines, which consider target populations with cultures different from the original population (39). The translation was independently conducted by two university teachers with strong English-language proficiency and several years of experience in academic research. Both translators had experience in scholarly research and were familiar with the conceptual interpretation of research instruments and academic terminology. The translators are not authors of this manuscript and have never seen the original scale, thus preventing a conflict of interest and presumption of the original scale.

**Figure 1:**
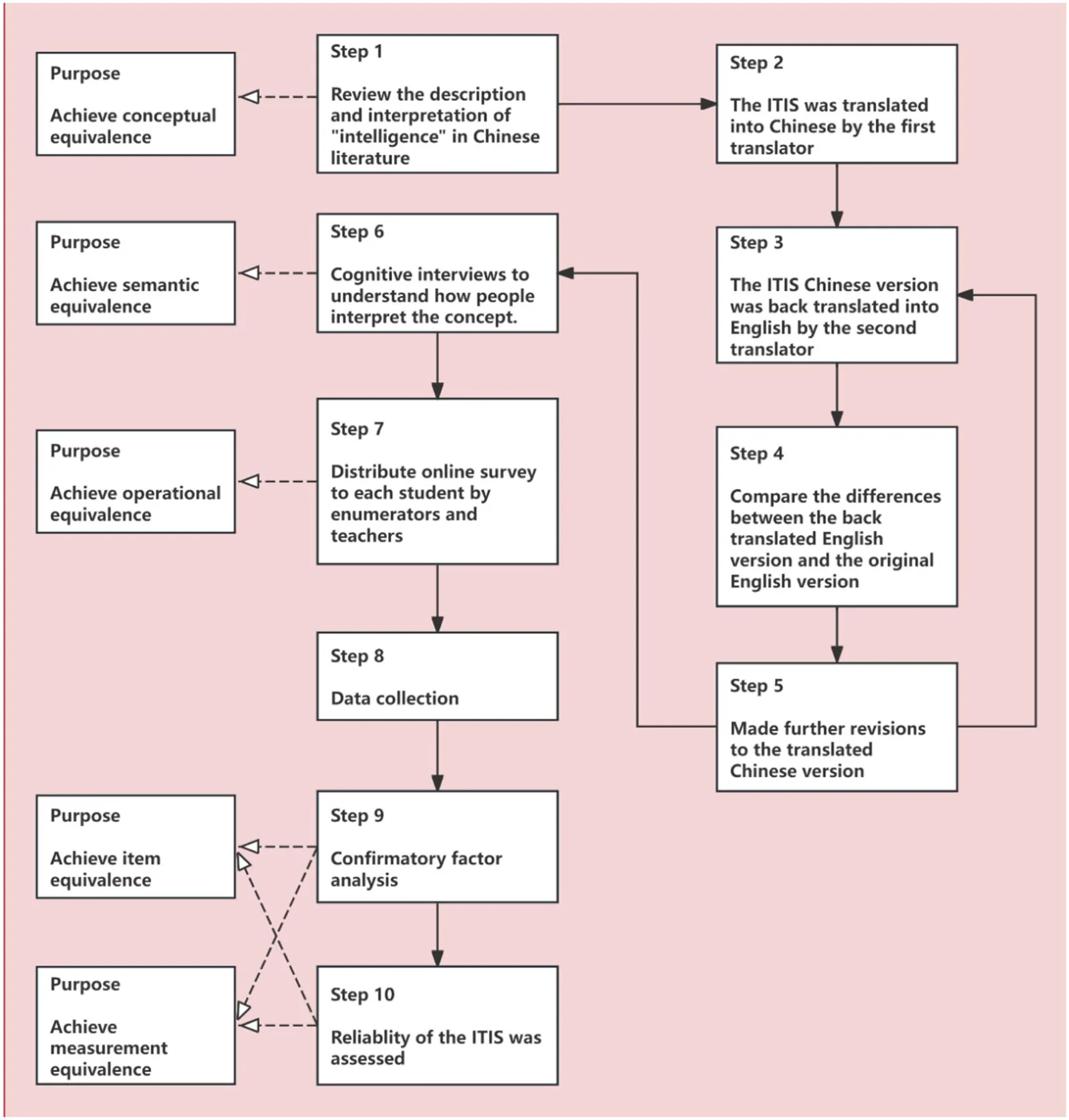
Procedure to achieve functional equivalence in cross-cultural translation.

The English version of the ITIS was translated into Chinese by the first translator. Next, the Chinese version of the ITIS was independently back-translated into English by the second translator. The first author and translator then compared the back-translated English version and the original English version of the ITIS. The first translator made further revisions to the translated Chinese version and resubmitted it to the second translator. The process was repeated until irreconcilable differences arose. When the back-translation ended, a joint meeting was held between the first author (who understood the research context) and the two translators (who understood the language). They examined the deviations in meaning between the original and translated English versions of the ITIS and decided on appropriate Chinese terms to accurately convey the meaning of the original text.

### 2.5 Cognitive interview

Thorough cultural adaptation is necessary when translating a questionnaire for use in a new setting rather than simply providing a verbal translation (35). One technique that can provide detailed insights into potential obstacles or deficiencies in adapting a questionnaire to different cultural contexts is the cognitive interview (40, 41).

In our study, individual cognitive interviews were conducted with five medical students from Fujian Medical University. Before the interviews, all participants were thoroughly informed of the study objectives, the voluntary nature of their participation, the fact that their statements would be recorded, and the assurance of anonymity. Informed consent was obtained from all the participants.

During the cognitive interviews, participants were instructed to “think aloud” as they responded to the ITIS (41). Throughout the process, the first author asked retrospective probing questions and recorded participants' answers and important viewpoints by writing them on paper. These probing questions varied in scope, ranging from general inquiries (e.g., “What thoughts crossed your mind when answering this question?”) to specific queries (e.g., “What concepts are evoked when you think about “intelligence’ in this context?”) to more technical probes (e.g., “Could you explain why you selected the answer for this question?”). These questions were primarily intended to clarify the participants' understanding and perspectives.

After each cognitive interview, the translated Chinese version of the ITIS was corrected and displayed to the next participant. This process was repeated until participants made no further recommendations.

### 2.6 Online survey

Participants were recruited using a convenience sampling approach with snowball distribution. The Chinese version of the ITIS was administered online through Wenjuanxing, a commonly used online survey platform in China. The electronic questionnaire invitation was initially distributed to class counsellors at participating medical schools, who subsequently disseminated the invitation to eligible students through WeChat. Students were encouraged to share the questionnaire to other students and completed the questionnaire independently online. Before starting the online survey, participants were informed about the study's objectives and data collection procedures, and that personal information, such as name and age, would be collected to conduct the test-retest. Participants were also assured of participation on a voluntary basis and that they had the right to withdraw from the study at any time without facing consequences. Data were collected between September and November 2023. Following data collection, the dataset was screened for completeness and duplicate submissions. Incomplete questionnaires were excluded from the final analysis, and duplicate online responses were identified and removed to ensure that each participant contributed only one response to the dataset.

### 2.7 Test-retest

Reliability refers to the consistency and stability of measurements over repeated assessments. Test–retest reliability of the Chinese version of the ITIS was evaluated separately for the Fixed Mindset and Growth Mindset dimensions using intraclass correlation coefficients (ICCs).

### 2.8 Data analysis

Descriptive statistics were used to summarize the characteristics of the participants, including age and gender. Construct validity, convergent validity, and discriminant validity of the Chinese version of the ITIS were evaluated using confirmatory factor analysis (CFA) in IBM SPSS AMOS 26.

For construct validity, a two-factor measurement model was specified, with Items 1–4 loading on the Fixed Mindset factor and Items 5–8 loading on the Growth Mindset factor. Model fit was evaluated using the chi-square statistic (*χ*^2^), the chi-square-to-degrees-of-freedom ratio (CMIN/DF), the normed fit index (NFI), relative fit index (RFI), incremental fit index (IFI), Tucker–Lewis index (TLI), comparative fit index (CFI), standardized root mean square residual (SRMR), and root mean square error of approximation (RMSEA). A CMIN/DF value below 3 was considered indicative of good fit, whereas a value below 5 was considered acceptable (42, 43). For the incremental fit indices, values above 0.90 were considered acceptable (42, 44). An SRMR value below 0.08 and an RMSEA value below 0.08 were considered indicative of acceptable model fit (42, 45, 46). Standardized factor loadings of 0.70 or higher were considered satisfactory.

Modification indices were examined as a diagnostic procedure during CFA. Potential cross-loadings were evaluated against the theoretically specified two-factor structure and the content of the corresponding items. Parameters were not added solely on the basis of modification indices. Where residual covariances were considered, they were evaluated in conjunction with the semantic content of the relevant items and their potential to represent item-level shared variance beyond the common latent factor (47).

Convergent validity was assessed using standardized factor loadings, composite reliability (CR), and average variance extracted (AVE). Standardized factor loadings of 0.70 or higher, CR values of 0.70 or higher, and AVE values of 0.50 or higher were considered evidence of satisfactory convergent validity.

Discriminant validity was evaluated using the Fornell–Larcker criterion, maximum shared variance (MSV), and the heterotrait–monotrait (HTMT) ratio of correlations. According to the Fornell–Larcker criterion, the square root of the AVE for each construct should exceed its correlation with other constructs. In addition, MSV should be lower than AVE for each construct. An HTMT value below 0.90 was considered indicative of adequate discriminant validity.

A two-way mixed-effects model with absolute agreement was used, and single-measure ICC estimates with 95% confidence intervals were reported because the reliability of a single administration of the questionnaire was of interest (48). Cronbach's alpha was also calculated separately for the two dimensions to assess internal consistency.

## 3 Results

### 3.1 Translation of the implicit theories of intelligence scale

The back-translated and original English versions of the ITIS were compared and several minor wording differences were identified. During the forward translation, “intelligence” was translated into “智商” (Chinese pronunciation: “zhi-shang”), which means Intelligence Quotient; this term is more widely used in the Chinese language context. To ensure that respondents could understand the difference between scoring options of “3 = mostly agree, 4 = mostly disagree”, “mostly” was translated into “部分” (Chinese pronunciation: “bu-fen”), which means partly.

### 3.2 Cognitive interview

During the interview, the first student felt confused. He said according to the introduction, he thought the questionnaire was not focused on the evaluation of himself but on the general situation of the public, which led to his hesitation in answering. He explained that people of different ages may have different understandings of or beliefs about intelligence. Subsequently, based on the interview results of the first responder, “your” was added to the introduction of the questionnaire: This questionnaire is designed to investigate your ideas about intelligence (Table 1).

**Table 1:** The revised translation after cognitive interview.

Translation stage	Wording
Original wordings	This questionnaire has been designed to investigate ideas about intelligence.
Initial translation	本问卷旨在调查人们对智商的想法。
The meaning of initial translation in English	This questionnaire has been designed to investigate peoples’ ideas about intelligence.
Revision made	Changed “peoples’” into “your”
Revised translation	本问卷旨在调查你对智商的想法。
The meaning of revised translation in English	This questionnaire has been designed to investigate your ideas about intelligence.

The revised translation was tested in the second cognitive interview, and no additional concerns were raised. Subsequently, the third, fourth, and fifth interviews did not raise additional concerns. All participants claimed that each question was understandable and simple to answer. They confirmed the clarity of the instructions and questionnaire format. Hence, the interviews were stopped, and the Chinese version of the ITIS was finalized.

### 3.3 Respondents' characteristics

A total of 444 completed questionnaires were received from medical students through an online survey, providing an adequate sample size for psychometric validation and subsequent analyses. Because the questionnaire was distributed through this indirect snowball approach, the exact number of students who received or viewed the invitation could not be determined. Notably, the participant group was evenly split, with 222 female and 222 male students. The participants' average age was 22.3 years (*SD* = 0.85; range = 20–25 years).

### 3.4 Validity analyses

Prior to CFA, the distributional characteristics of the eight ITIS items were examined using skewness and kurtosis. The item-level skewness values ranged from −0.452 to 0.802, and kurtosis values ranged from −0.715 to 0.481, indicating relatively limited deviations from univariate normality. However, the multivariate kurtosis was 45.748 (critical ratio = 38.104), indicating that the assumption of multivariate normality was not fully satisfied. Given the sample size of 444 and the relatively limited item-level skewness and kurtosis, CFA was conducted using maximum likelihood estimation in IBM SPSS AMOS 26. The model-fit indices were interpreted together rather than relying solely on the chi-square statistic.

CFA was conducted to assess the construct validity of the model. The factor loadings for each item ranged from 0.78 to 0.91 (Figure 2). The goodness-of-fit statistics for the model were as follows: *χ*^2^ = 52.293, *df* = 17, CMIN/DF = 3.076, CFI = 0.984, SRMR = 0.059, RMSEA = 0.068, NFI = 0.977, RFI = 0.962, IFI = 0.984, TLI = 0.974, parsimony-adjusted comparative fit index (PCFI) = 0.598 (*p* < 0.05), indicating satisfactory model fit and support for the construct validity of the Chinese version of the ITIS (Table 2).

**Figure 2:**
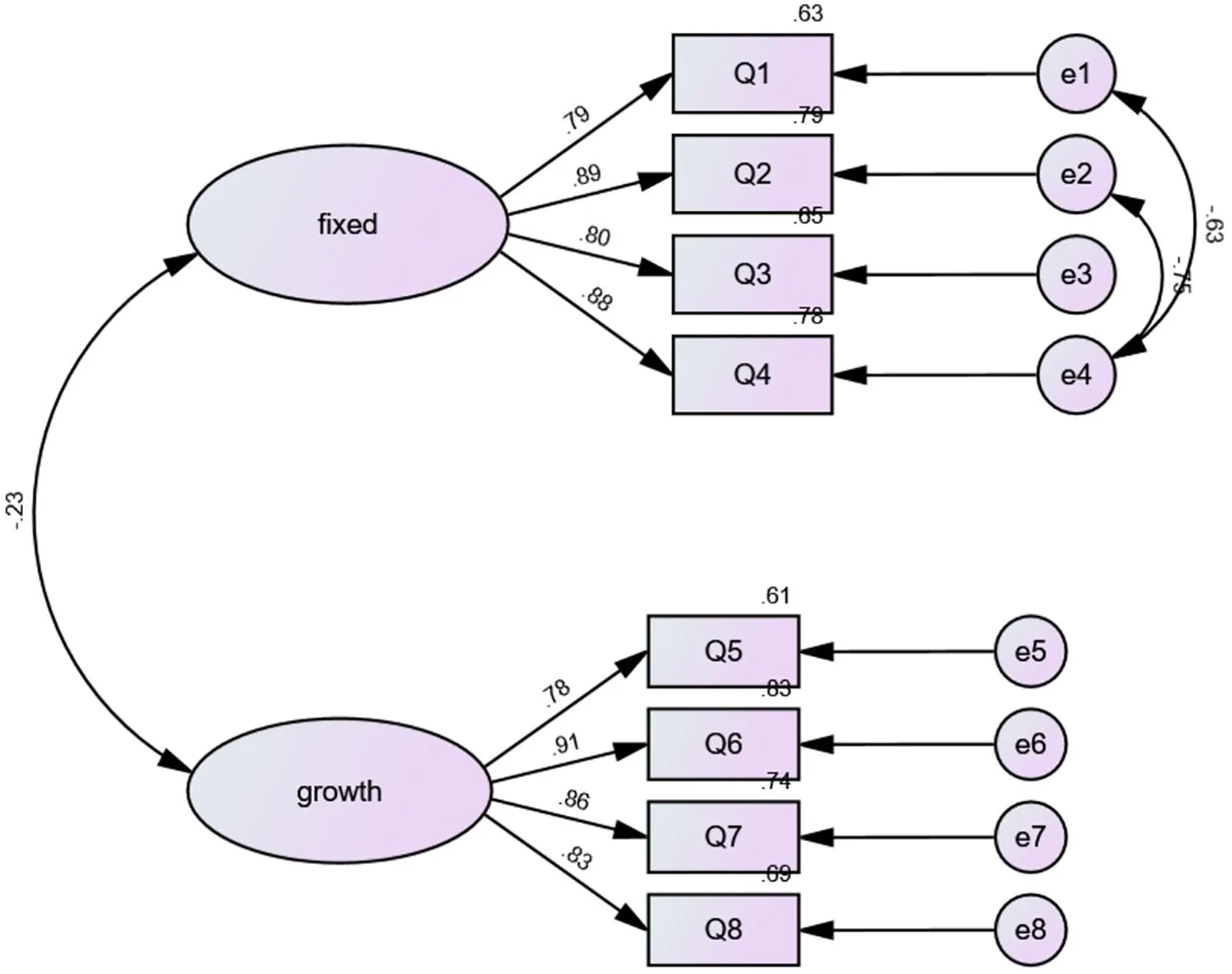
Schematic diagram of standardized model fitting of ITIS-Chinese version (*N* = 444).

**Table 2:** The fitting indexes of confirmatory factor analysis of the Chinese version of ITIS (*n* = 444).

Index	Criteria	Results of the Chinese ITIS	Evaluation
*X* ^2^		52.293	
*df*		17	
*X*^2^/df	<5	3.076	Acceptable
CFI	>0.95	0.984	Acceptable
SRMR	<0.08	0.059	Acceptable
RMSEA	<0.08	0.068	Acceptable
NFI	>0.90	0.977	Acceptable
RFI	>0.90	0.962	Acceptable
IFI	>0.90	0.984	Acceptable
TLI	>0.90	0.974	Acceptable

*N* = 444. ITIS, Implicit Theories of Intelligence Scale; CFI, comparative fit index; SRMR, standardized root mean square residual; RMSEA, root mean square error of approximation; NFI, normed fit index; RFI, relative fit index; IFI, incremental fit index; TLI, Tucker–Lewis index.

For convergent validity, the average variance extracted (AVE) values for the Fixed Mindset and Growth Mindset factors were 0.713 and 0.717, respectively. Both exceeded the recommended threshold of 0.50, indicating that each latent construct accounted for more than 70% of the average variance in its respective indicators. The composite reliability (CR) values were 0.908 and 0.910, respectively, both exceeding the recommended threshold of 0.70 (Table 3). Together with the satisfactory standardized factor loadings, these findings supported adequate convergent validity.

**Table 3:** Composite reliability, AVE, MSV and HTMT analysis of the Chinese version of ITIS (*n* = 444).

Factor	CR	AVE	MSV	fixed	growth
fixed	0.908	0.713	0.052	0.844	
growth	0.910	0.717	0.052	−0.228	0.847
				**HTMT Analysis**	
fixed				—	—
growth				0.217	—

*N* = 444. CR, composite reliability; AVE, average variance extracted; MSV, maximum shared variation; HTMT, Heterotrait–monotrait ratio of correlations; ITIS, Implicit Theories of Intelligence Scale.

For discriminant validity, the square root of the AVE for each construct was greater than the correlation between the Fixed Mindset and Growth Mindset factors (Table 2), satisfying the Fornell–Larcker criterion. In addition, the maximum shared variance (MSV) values were lower than the corresponding AVE values. The heterotrait–monotrait (HTMT) ratios were also below 0.90. Collectively, these findings provided evidence of adequate discriminant validity.

### 3.5 Reliability analyses

The internal consistency and test–retest reliability of the two ITIS dimensions were assessed separately. The Fixed Mindset dimension demonstrated acceptable internal consistency, with a Cronbach's alpha of 0.793. Its test–retest reliability was moderate, with a single-measure ICC of 0.662 (95% CI: 0.443–0.806, *p* < 0.001). The Growth Mindset dimension demonstrated good internal consistency, with a Cronbach's alpha of 0.875. However, its test–retest reliability was limited, with a single-measure ICC of 0.159 (95% CI: −0.158–0.446, *p* = 0.162). These findings indicate that the two dimensions demonstrated different levels of temporal stability in the test–retest sample.

### 3.6 Characteristics of intelligence mindset among fifth-year Chinese medical students

The descriptive statistics of fixed and growth mindset items are presented in Table 4 and Figure 3. For fixed mindset items, the mean scores ranged from 2.64 (SD = 1.09) to 2.86 (SD = 1.56). Given that lower scores indicate stronger endorsement of fixed mindset beliefs, these findings suggest that participants tended to endorse beliefs consistent with a fixed view of intelligence. For growth mindset items, the mean scores ranged from 3.81 (SD = 1.16) to 3.96 (SD = 1.21), indicating relatively lower endorsement of beliefs that intelligence can be developed through effort and learning. Overall, the findings suggest that final-year Chinese medical students demonstrated a tendency toward fixed mindset orientations.

**Table 4:** Descriptive statistics of the 8 items among Chinese final-year medical students (*n* = 444).

Domain	Item	Mean	SD
Fixed mindset	F1: You have a certain amount of intelligence, and you can't really do much to change it.	2.69	1.12
	F2: Your intelligence is something about you that you can't change very much.	2.74	1.11
	F3: To be honest, you can't really change how intelligent you are.	2.86	1.56
	F4: You can learn new things, but you can't really change your basic intelligence.	2.64	1.09
Growth mindset	G1: No matter who you are, you can significantly change your intelligence level.	3.83	1.21
	G2: You can always substantially change how intelligent you are.	3.96	1.20
	G3: No matter how much intelligence you have, you can always change it quite a bit.	3.81	1.16
	G4: You can change even your basic intelligence level considerably.	3.89	1.23

Items were rated on a six-point Likert scale ranging from 1 (strongly agree) to 6 (strongly disagree). Higher scores indicate lower endorsement of mindset beliefs, whereas lower scores indicate stronger endorsement of mindset beliefs.

**Figure 3:**
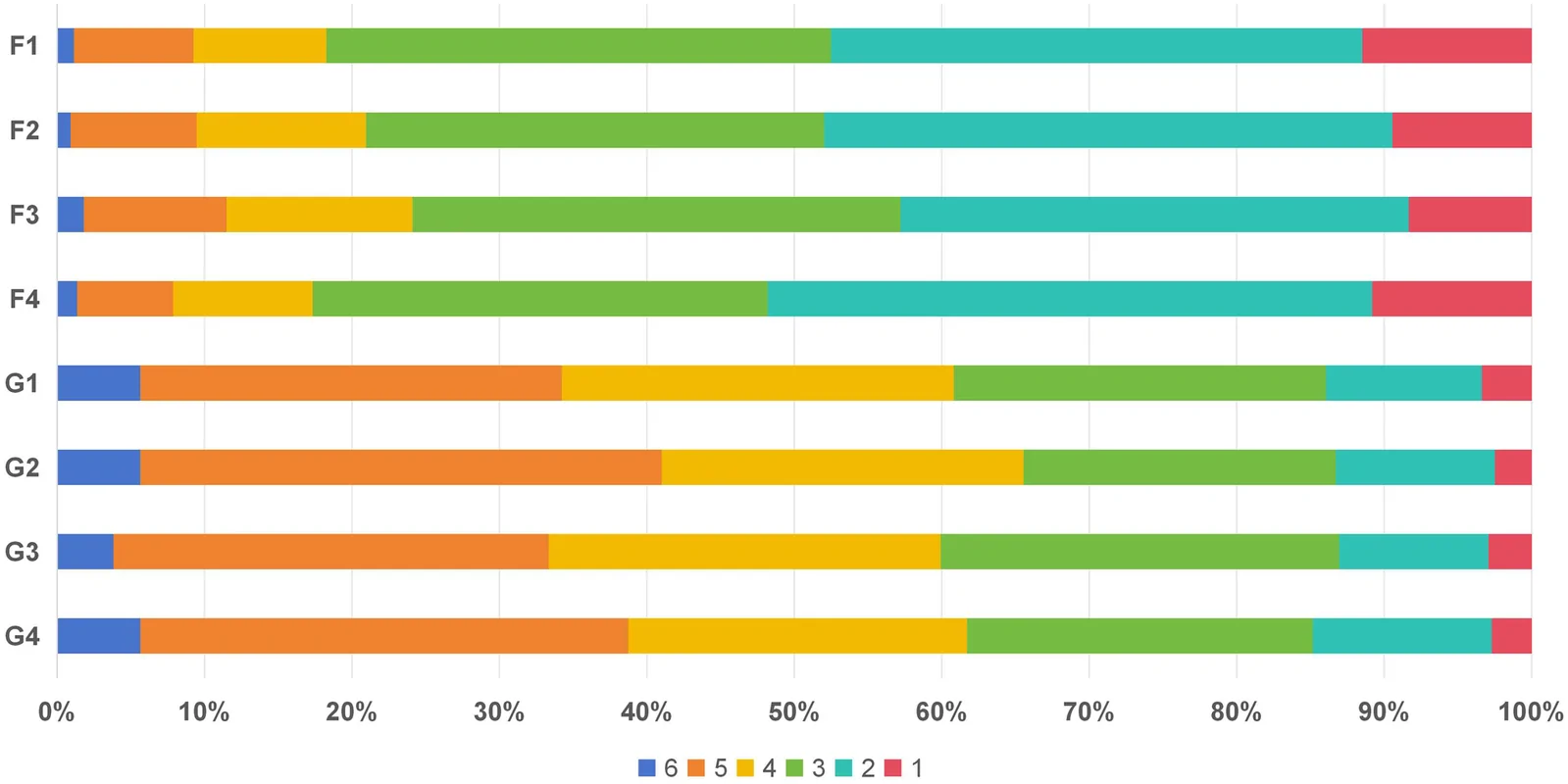
Characteristics of intelligence mindset Among fifth-year Chinese medical students. Note. Items were rated on a six-point Likert scale ranging from 1 (strongly agree) to 6 (strongly disagree). Higher scores indicate lower endorsement of mindset beliefs, whereas lower scores indicate stronger endorsement of mindset beliefs.

## 4 Discussion

Our study aimed to translate and validate the Chinese version of the ITIS. To establish conceptual and semantic equivalence, we employed a back-translation technique using two independent translators and cognitive interviews. In the process of back-translating the ITIS, the translation of the term “intelligence” emerged as a fundamental step. Participants' understanding of key terms in the questionnaire is critical as it directly impacts the accuracy and reliability of the survey results. Its use can be influenced by cultural, societal, and political factors, leading to diverse interpretations and perceptions; hence, it has drastically varied meanings for different individuals. It has been previously conjectured that these variations in intelligence comprehension may have considerable effects on the assessment of intelligence mindset (49). Cultural differences have also been found to be related to how students define intelligence mindset. Most Chinese students believe that intelligence is a fluid skill, whereas American students were more likely to describe intelligence as a consolidated competency (50). Based on the results from previous research and the understanding of this term in Chinese culture, we chose the term “智商 (Zhi-shang)” instead of “智力 (Zhi-li),” which has a similar meaning to “IQ” in English. This choice is intended to eliminate some ambiguities surrounding the interpretation of the term, particularly regarding its meaning of innate cognitive abilities. During the cognitive interviews, most students agreed with this translation. By ensuring that every participant understood the key terms, researchers can collect more accurate and trustworthy survey data. This study adopted a six-step process of forward-backward translation and cognitive interviews, systematically addressing the risks of semantic ambiguity and conceptual deviation in previous Chinese versions of the ITIS.

After translation, we examined the validity and reliability of the newly translated Chinese version of the ITIS. The results showed that the scale had strong test-retest reliability at four weeks and good composite reliability. CFA was additionally employed to examine the two factors that comprise the ITIS' overall structure. In empirical research, fixed-fit cutoffs are frequently used to detect possible model misspecifications and select a condensed model (51). The best method to determine the quality of fit is to consider various viewpoints, as recommended by Marsh (52). To assess the model fit, it is usually advised to look at several qualitative indices with proven characteristics. Several indices were used to verify the Chinese version of the ITIS: *χ*^2^/*df*, CFI, SRMR, RMSEA, RFI, IFI, TLI, NFI, IFI, and PCFI. Each index met the required threshold (52). The CFA results suggests that the updated Chinese version of the ITIS has excellent construct validity, as the modified two-factor model is assumed to better match the data.

Our study also provides strong evidence for the use of the two-factor structure of the ITIS. According to Dweck *et al*. (53), “believing that something cannot be changed is the logical opposite of believing it can be changed,” which characterizes fixed and growth mindsets as being at opposing ends of the spectrum (2). Costa and Faria (54) conducted a literature review and discovered that self-report measures are always used to test attitudes toward intelligence, and most of these scales use the ITIS by Dweck (53). The two subscales are frequently merged into a single measure when growth and fixed items are included. The issue becomes even more confusing when these fixed mentality-only elements are then reverse-coded and given the title “growth mindset” (55). Some studies have determined strong negative correlations between growth and fixed subscales, which may support their use as a single construct. However, fixed and growth mindsets were moderately negatively correlated, suggesting that although these two mindsets are related, it is important for future researchers to measure the levels of both dimensions (56). In our study, the correlation between growth and fixed subscales is small and negative (*r* = −0.23). Therefore, we suggest the use of two-factor structures to measure intelligence mindset in future research to fully reflect an individual's true ideas about their level of intelligence.

Language and culture are closely interrelated. Cultural norms and values regarding intelligence played crucial roles in shaping individuals' attitudes during the study. Furthermore, students with different understandings of the item “intelligence” yielded varying growth mindset scores. Students who viewed intelligence as having the same meaning as knowledge tended to have a growth mindset according to the ITIS (53). Based on this, we postulate that culture might influence this mindset. Hence, future studies should consider cultural factors and investigate their impact on how students define and perceive intelligence. Therefore, understanding and appreciating cultural differences is essential for educators and policymakers to create inclusive learning environments that accommodate diverse perspectives and promote positive attitudes toward intelligence and learning. If Chinese medical students exhibit a comparatively less than ideal performance in this aspect, it indicates that the cultivation of a growth mindset should be emphasized in modern Chinese medical education (58).

The present study provides preliminary evidence that mindset orientations among medical students may vary across different cultural and educational contexts. In our study, Chinese medical students demonstrated relatively stronger endorsement of fixed mindset beliefs, whereas previous studies conducted in Western contexts have generally reported stronger growth mindset orientations among medical students. For example, Richardson et al. (19) examined 168 medical students in the United States using the ITIS and reported high levels of growth mindset endorsement (Mean = 1.68), suggesting that students tended to view intelligence as developable and perceived challenges as opportunities for learning (22). Although direct comparisons should be interpreted cautiously due to differences in educational systems, cultural backgrounds, and measurement contexts, these findings suggest that medical students' beliefs about intelligence may be shaped by broader sociocultural environments.

The relatively stronger fixed mindset orientation observed among Chinese medical students may be related to the characteristics of Confucian Heritage Culture (CHC) and its interaction with contemporary educational practices. Confucian values traditionally emphasize learning, self-improvement, perseverance, and the importance of effort, which are conceptually consistent with growth-oriented beliefs. Within this cultural framework, personal development is often viewed as a continuous process achieved through discipline and persistent learning. Therefore, CHC should not be considered inherently associated with fixed mindset beliefs.

Recent cross-cultural research supports the view that cultural influences on mindset are complex rather than unidirectional. For example, studies comparing Chinese and Western students have suggested that students from different cultural backgrounds may conceptualize intelligence, effort, and success in distinct ways (50). Similarly, research from other Asian contexts, such as Malaysia, has demonstrated that medical students may show relatively stronger growth mindset tendencies despite sharing some collectivistic cultural characteristics, suggesting that cultural values interact with educational environments rather than independently determine mindset development. Understanding this complex interaction between cultural values and educational experiences may provide valuable insights for developing culturally responsive strategies to foster adaptive mindsets among medical students.

## 5 Limitations

Several limitations should be considered when interpreting the findings of this study. First, the participants were recruited using a convenience-based online sampling approach and were limited to final-year undergraduate medical students in Fujian Province. Therefore, the findings may not be generalisable to medical students from other regions of China, other stages of medical education, or other student populations. Online recruitment may also have introduced selection bias, as students who chose to participate may have differed from nonresponders in ways that were not measured. Moreover, information regarding the number and characteristics of nonresponders was unavailable, limiting the assessment of potential nonresponse bias.

Second, although cognitive interviews were conducted to evaluate the clarity and comprehensibility of the translated items, these interviews were conducted at a single institution. Consequently, the range of linguistic and contextual perspectives represented during the cognitive interviewing process may have been limited.

Third, the test–retest analysis was conducted in a relatively small subsample of 40 participants. The resulting ICC estimates, particularly for the Growth Mindset dimension, were associated with relatively wide confidence intervals. The limited test–retest sample therefore warrants caution when interpreting the temporal stability of the translated scale.

Fourth, the study relied on self-reported questionnaire responses, which may be subject to response and social desirability biases. Finally, criterion validity was not examined against objective behavioural, academic, or educational outcomes. Future studies should therefore evaluate the performance of the Chinese ITIS in larger and more diverse samples and examine its relationships with relevant behavioural and educational outcomes.

## 6 Conclusions

This study provides evidence supporting the construct validity, convergent validity, discriminant validity, internal consistency, and test–retest reliability of the Chinese version of the Implicit Theories of Intelligence Scale within the present sample of final-year undergraduate medical students in Fujian, China. The findings also indicated relatively stronger endorsement of fixed-mindset beliefs than growth-mindset beliefs in this sample. However, the findings should not be interpreted as definitive validation of the instrument for all Chinese medical students. Further research using larger and more diverse samples, multi-institutional recruitment, and external behavioural or educational criteria is warranted to further evaluate the validity, reliability, and applicability of the Chinese ITIS across different medical education contexts.

## Data Availability

The raw data supporting the conclusions of this article will be made available by the authors, without undue reservation.
